# Knowledge of young African American adults about heart disease: a cross-sectional survey

**DOI:** 10.1186/1471-2458-11-248

**Published:** 2011-04-19

**Authors:** Donna M Winham, Kathleen M Jones

**Affiliations:** 1 Nutrition Program, College of Nursing and Health Innovation, Arizona State University, Mesa, Arizona, USA; 2 Arizona Nutrition Network, Arizona Department of Health Services, Phoenix, Arizona, USA

## Abstract

**Background:**

African Americans have higher rates of cardiovascular disease (CVD) mortality than other ethnic groups. Young adults are prime targets for intervention strategies to prevent and reduce disease risk. The study purpose was to determine the level of knowledge of lifestyle risk factors for CVD among young African American adults in Phoenix. The results will be used to guide the development of CVD outreach programs targeted to this population. The Health Belief Model was used as a conceptual framework.

**Methods:**

A convenience sample of 172 African American men and women aged 18-26 years completed a questionnaire adapted from the American Heart Association national surveys. Descriptive statistics were compared by age, gender, education level, and health status variables including BMI, smoking status, and physical activity.

**Results:**

Some aspects of heart-disease were well known among young adult African Americans. Knowledge of certain other important risk factors (menopause) and preventive behaviors (eating fewer animal products), however, was more variable and inconsistent among the respondents. Differences in knowledge of individual variables was greater by education level than by gender overall. Predictors of a summary CVD knowledge score included higher education, female gender, and high self-efficacy (adjusted R^2 ^= 0.158, *p *< .001). Predictors of self-efficacy in changing CVD risk were higher education and perceived low risk of CVD (adjusted R^2 ^= 0.064, *p *< .001), but these characteristics explained only 6% of the variance.

**Conclusions:**

Evaluation of baseline knowledge of CVD is essential before designing and implementing health promotion programs. Existing strengths and weaknesses in knowledge can guide tailoring of programs to be more effective. Further research would help to identify the range of other characteristics that determine knowledge and risk perception.

## Background

Heart disease is the leading cause of death in the United States, claiming approximately 700,000 lives-nearly 30% of all United States (US) deaths-each year [1]. African Americans constitute 12.8% of the US population, yet their risk of developing cardiovascular disease (CVD) is three times greater than Whites and they also have twice the risk of mortality [2,3]. To complicate matters further, African Americans have higher premature CVD deaths than Whites and may have earlier onset of disease [4,5].

Differences in access to and quality of health care, dietary intakes, lifestyle factors (e.g., smoking), neighborhood characteristics, socioeconomic status, sociocultural attitudes toward disease, persistent racial discrimination, and genetics have been offered as explanations for poor CVD health outcomes among African Americans [6,7]. Yet not all of these factors contribute to CVD risk for every African American. Many disease risk factors are amenable to change, and all individuals can reduce their risk of CVD morbidity and mortality, especially if healthful behaviors are established in childhood or changed in young adulthood.

Knowledge of CVD risk factors is essential for a person to make an informed decision about engaging in or continuing certain behaviors that may increase disease risk, such as smoking, not exercising, or consuming high-fat foods [8]. The Health Belief Model (HBM) suggests that a person must feel susceptible to the disease in order to change his or her behaviors [9,10]. Thus, there must be perceived risk or some willingness to change for many individuals to alter behaviors. For behavior change to occur, an individual must be aware of the potential negative consequences of his or her current actions. In this sense, knowledge alone may be insufficient to cause behavior change, and without a perception of self-risk, there is little impetus to change [10,11].

The successful development and implementation of a health promotion program, depends upon identification of the scope and breadth of baseline knowledge among the targeted group members [12]. For example, regional differences in beliefs and knowledge may exist. If programs are to be culturally appropriate and salient for the target audience, baseline information is essential before investing in new program development or adapting existing programs in a given region [13].

Most researchers have used national surveys [14] or surveys undertaken in the rural South [7,15] to study CVD risk knowledge among African Americans. National surveys assume ethnic group homogeneity and can miss local details such as the specific and unique characteristics of a certain population. African Americans are a heterogeneous group in terms of income, education, cultural affiliation, religiosity, and life experiences [16]. Although more than 50% of African Americans live in the southern US, one cannot assume these individuals' characteristics are representative of African Americans living in other regions where the political, economic, and social environments are different [17]. In fact, CVD mortality is higher in the southeastern states for people of all races and ethnicities [18]. The African American experience in Arizona may be different because of the history of political and social organizations in the region and the distance, both in time and geography, from the lifestyle behaviors of the rural South [19]. Knowledge about CVD may differ as well. Nevertheless, despite recommendations that providers determine baseline knowledge before applying evidence-based guidelines on health promotion with communities in other settings and locations, intervention programs are not always tailored to fit the community of interest [20]. This can result in poor intervention outcomes and wasted efforts.

CVD prevention and intervention research often focuses on middle-aged adults with the signs and symptoms of disease. These individuals may have greater self-efficacy and motivation to modify behavior that reduces CVD risk than their adult peers who do not have CVD [21,22]. Moreover, health-promotion and prevention efforts, if started early enough, can have a greater impact on quality of life than therapeutic interventions alone when enacted at a more advanced age [23].

For young adults, behavior patterns and trajectories established now will influence their health for a lifetime [24]. Young adults are at a critical transition period in their lives. They establish adulthood health behaviors, move from their parents' home to live independently, and, for some, begin having children during this life stage [24]. Behavior patterns or decisions about lifestyle choices (e.g., dietary intakes, physical activity, and smoking) made in early adulthood initiate health trajectories that predict chronic-disease risk, including CVD, later in life [25-27]. Furthermore, individuals who become parents may sway the health behaviors of the next generation by the examples they set in diet and lifestyle behaviors. The intergenerational effects are amplified because many young adults are involved with the care of their parents or grandparents as well as their own children. Thus, young adults may be strategically poised to influence health behaviors throughout all stages of the life cycle [28].

Tailoring health messages ensures that the target group receives information that is salient to their concerns and situation. If people hear the same message they already know (e.g., high cholesterol is bad), but they do not know how to act on this information or perceive it as irrelevant, they may dismiss the message [17]. Process evaluation or crosschecking to determine if messages are understood correctly and appropriate actions are taken is important after program initiation as well.

African Americans are 4.3% of Arizona's total population, yet their CVD mortality rate is higher in the state than it is among other ethnic/racial groups [29,30]. In 2007, African Americans living in Arizona had a 60% greater risk of dying from CVD than Arizona's Asian population, which had the lowest risk of death due to heart disease [29]. As a small and geographically spread-out minority, targeted outreach efforts to improve health and reduce CVD risk for African Americans are needed [17].

The HBM, which focuses on an individual's perceptions about a disease state or condition, guided the study design and research question development. The HBM states that for behavior change to occur individuals must perceive a disease or condition as serious (perceived seriousness), consider themselves vulnerable to developing the disease (perceived susceptibility), see positive outcomes associated with the desired behavior change (perceived benefits) and no obstacles to changing behavior (perceived barriers). Knowledge about a disease and risk as well as confidence in one's ability to take all the steps associated with behavior change (perceived self-efficacy), added to the model as it was developed, is also now an integral part of the HBM. These key concepts are collectively referred to as attitudes in the current study [9].

The research objectives were to (1) describe knowledge, attitudes, and practices about CVD among young adult African Americans in metropolitan Phoenix and (2) determine if perceived seriousness, perceived susceptibility, perceived self-efficacy and perceived benefits related to CVD risk varied by gender, age, education, and health status.

## Methods

### Study design and population

The design was a cross-sectional descriptive study. A convenience sample of young adult men and women who self-identified as having African ancestry was recruited. For the purpose of the study, a young adult was defined as a person between the ages of 18 and 29 years [31]. Targeted recruitment efforts were made to include participants from all socioeconomic and educational backgrounds, including those who were in the Special Supplemental Nutrition Program for Women, Infants, and Children (WIC), African American churches, campus groups, and local community organizations. Data were collected between May and December 2007. During preliminary analysis participants aged 27-29 (n = 24) were excluded from further analysis because almost all of them were college educated (22/24). Any observed associations with the outcome measures and age would be driven by education alone. Therefore, retention of these older participants would have confounded observed associations because there were only 2 participants in this age group with less education. The final sample size consisted of 172 participants aged 18-26 years.

### Survey instruments and data collection procedures

A demographic questionnaire was developed that included questions about age and education. With permission from the American Heart Association (AHA) a national survey on women's knowledge, attitudes, and practices with regard to heart disease was used [14]. The questionnaire has been used in four national telephone surveys with women conducted by the AHA in 1997, 2000, 2003, and 2006 [14,32]. The survey asks about specific heart-disease domains such as health behavior awareness, knowledge of CVD risk factors, knowledge of symptoms and outcomes, treatment options, awareness of self-risk and preventive behaviors.

Several questions from the AHA questionnaire comprised the HBM conceptual framework. Perceived susceptibility was estimated using the statement "I am at low risk for a heart attack or stroke for a person my age" from the AHA questionnaire [14,32]. Self-efficacy was evaluated with the statement "There is nothing I can do to help prevent myself from getting heart disease." Both statements had four response categories, ranging from *agree strongly *to *disagree strongly*. Perceived seriousness of heart disease was assessed by two questions on the leading cause of death and on the outcomes of CVD. Perceived benefits were based on knowledge of disease outcomes and preventive behaviors. Cues to action were addressed through the number of risk factors and symptoms each person had (BMI, cholesterol, smoking, physical activity). Although part of the HBM, perceived barriers were not directly examined in this descriptive study because our focus was on baseline knowledge, not change.

The original AHA questionnaire targeted women aged 25 years or older. Consequently slight wording changes were made to address both male and female respondents and to convert the questionnaire to be self-administered rather than be delivered by an interview. Several questions about the use of hormone replacement therapy and chronic disease diagnosis were omitted because these topics were deemed less applicable to young adults. Initial pilot testing for content validity after these minor modifications was done with a convenience sample of nine African American men and women aged 21-32. For pilot testing, African American ethnicity was more important than the exact age of the convenience sample. Minor formatting changes were made before official data collection.

Most participants contacted the researchers in response to email or listserv advertisements of the study (117/172). After the initial contact with a participant by email or telephone, the researcher arranged to meet at a mutually convenient time and public location to complete the survey (e.g., local library or community center). Three did not show up for scheduled appointments, and one declined to complete the survey. The other 55 participants were convenience samples from three churches and two WIC clinics. At these sites, the researcher set up with a table and participants approached her for more information. We were unable to track the number of people who declined to participate because these were convenience samples at these sites. Participants received a $15 gift card to a major retailer after they completed the survey. Participants provided written, informed consent, and all procedures were approved by the Arizona State University Research Compliance Office Institutional Review Board.

### Data management and transformations

Data entry and cleaning were ongoing over the course of the study. The continuous variables of age and education were correlated. To reduce confounding effects, two dichotomous variables were created based on logical breaks in the sample distributions. Age was dichotomized at the approximate 50^th ^cumulative percentile into two similarly sized groups of those aged 18-22 years and those aged 23-26 years. The bivariate education variable was split between those without a college education and those with any completed years of college.

A composite measure of health status risk was created by summing the presence or absence of risk behaviors. Points were assigned as follows: 1 point for current smoking, 1 point for body mass index (BMI) in overweight or obesity range, 1 point for not exercising four or more times per month. For analysis, these scores were categorized into tertiles of 0 risk factors, 1 risk factor, and 2+ risk factors.

Three summary knowledge index variables were created to use in multiple regression analysis to test the research hypotheses. The scores for the 12-item true-false questions on understanding of heart disease, the 6-item question of knowledge concerning heart attack warning signs, and the 5-item question of knowledge concerning stroke warning signs were coded to reflect 1 point for each correct answer and 0 points for incorrect answers. Each section was summed individually to create three separate indices: knowledge of heart-disease, of the warning signs of heart attack, and of the warning signs of stroke [10,33].

### Statistical analysis and modeling

Univariate relationships of continuous variables were analyzed between age and gender groups by independent t-tests. Chi-square was used to compare questionnaire variables by gender and education. Statistical significance was determined by *p *< 0.05. P-values below 0.05 are shown, but owing to multiple comparison we recommend that p-values < 0.05 but >0.01 should be regarded as trend values. Three multivariate linear regression models were tested to evaluate predictors of heart-disease knowledge, perceived susceptibility, and perceived self-efficacy. No adjustments were made for multiple comparisons and statistical tests.

## Results

### Demographics

A total of 172 adults aged 18-26 years old who self-identified as African American, Black or of African ancestry completed the study. Of the 172 participants, 36% were men, and 64% were women. Seventy-one percent (71%) had some college education or vocational training. Those with a college education were one year older on average than those without any college education. Statistically significant differences between genders were noted for education level, with fewer men than women having completed some college. Within education categories, those without college education had more children in the household. Table 1 shows the demographic characteristics for the sample classified by gender and college education.

**Table 1:** Demographic characteristics of young adult African American participants (N = 172)

Characteristics	Total	Male	Female	College	No college
		(n = 62)	(n = 110)	(n = 116)	(n = 56)
Age in years					
18-21	55.2	66.1	49.1	48.3	69.6
22-26	44.8	33.9	50.9	51.7	30.4
Years of Education					
College	71.4	56.5	73.6	---	---
No college	28.6	43.5^1^	26.4^1^		
Marital Status (%)					
Single/Divorced	86.0	83.7	84.5	85.2	89.3
Married/Live w/partner	15.5	11.3	15.5	14.8	10.7
Children under 18 years in household (%)					
Has 1+ child	23.8	6.5^2^	33.6^2^	16.5^3^	37.5^3^
No children	76.2	93.5	66.4	83.5	62.5
Household Income (%)					
Under $10,000/y	25.2	24.5	25.5	23.6	29.2
10-19,999	14.8	7.5	18.6	14.2	16.7
20-29,999	16.8	18.9	15.7	15.1	18.8
30-39,999	16.1	18.9	14.7	17.9	12.5
40-49,999	7.1	7.5	6.9	9.4	2.1
50-74,999	12.9	13.2	12.7	12.3	14.6
75- or more/y	7.1	3.8	2.9	2.8	4.2

All values are percentages. ^1 ^p = 0.021; ^2 ^p = 0.000; ^3^p = 0.003.

### Self-reported risk factors and preventive behaviors

Based on self-reported heights and weights, calculated BMI values indicated that 29.2% of the sample participants were overweight (i.e., BMI = 25.0 - 29.9) and 25.7% were obese (BMI ≥ 30.0; Table 2). The majority of participants had never smoked cigarettes. Current smoking was significantly lower among college-educated participants, than among those without any college education, but smoking did not differ by gender. More men than women reported exercising four or more times per week, Women were more likely than men to have had a cholesterol screen in the past 18 months). Respondents with college education were more likely than those without any college education to report their physicians had discussed heart disease with them. Almost 40% of the sample had two or more CVD risk factors (Table 2).

**Table 2:** Health behavior and risk factor characteristics of young adult African American participants (N = 172)

Characteristics	Total	Male	Female	College	No college
		(n = 62)	(n = 110)	(n = 116)	(n = 56)
BMI category (%)					
Underweight	1.8	0	2.8	2.6	0
Normal	43.3	40.3	45.0	41.4	47.3
Overweight	29.2	35.5	25.7	26.9	34.5
Obese	25.7	24.2	26.6	29.3	18.2
Exercise frequency (%)					
Almost never	16.3	9.7	20.0	18.1	12.5
Twice a month	14.0	6.5	18.2	16.4	8.9
Once a week	18.6	14.5	20.9	17.2	21.4
2-3 times per week	22.1	21.0	22.7	23.3	19.6
4+ times per week	29.1	48.4^1^	18.2^1^	25.0	37.5
Smoking frequency (%)					
Never smoked	70.9	69.4	71.8	76.7^2^	58.9^2^
Used to smoke, but quit	12.8	14.5	11.8	12.9	12.5
Smoke, but not every day	8.7	9.7	8.2	6.9	12.5
Smoke 1+ cigarettes/day	7.6	6.5	8.2	3.4	16.1
Risk factor summary score (%)					
No risk factors	20.9	22.6	20.0	22.4	17.9
One risk factor	44.2	53.2	39.1	41.4	50.0
Two+ risk factors	39.9	24.2	40.9	36.2	32.1
Cholesterol check in last 18 months (%)					
Yes	25.6	16.1^3^	30.9^3^	27.6	21.4
Blood pressure check past 18 months (%)					
Yes	83.7	82.3	84.5	83.6	83.9
Doctor discussed heart disease with you (%)					
Yes	20.9	14.5	24.5	25.9^4^	10.7^4^

*Note*. Data are %;; BMI definitions are: underweight ≤ 18.5, Normal 18.5-24.9, Overweight 25.0-29.9, and Obese ≥ 30.0. ^1 ^p = 0.001; ^2 ^p = 0.012; ^3 ^p = 0.033; ^4^p = 0.022.

### Perceived seriousness of heart disease

When asked as an open-ended question, "What is the leading cause of death?," 27% of the respondents stated obesity, followed by heart disease (16%), poor diet/unhealthy lifestyle (16%), HIV/AIDS (13%), cancer (11%), diabetes (6%), and other causes (11%). When given a list of options as a closed-ended question for the leading cause of death, heart disease was then reported most often (34%), followed by cancer (21%) and HIV/AIDS (12%). Awareness of heart disease as the leading cause of death was significantly higher among college educated (*p *= 0.003) and older respondents (*p *= 0.012). Most participants felt they were moderately well informed about heart disease among African Americans (51%). Slightly less (47%) felt they were well informed about stroke among African Americans. At the same time, 24% stated they were not at all informed about heart disease, and 33% said the same for stroke.

### Perceived susceptibility and risk

Almost 17% of the respondents strongly agreed with the statement "I am at low risk for a heart attack or stroke for a person my age." Another 43.6% agreed somewhat with the statement, but 39.5% either disagreed somewhat or disagreed strongly indicating they felt they were at risk. Other prevention and knowledge beliefs about heart disease are shown in Table 3. To determine their degree of perceived disease risk, participants were asked about their concerns of other diseases or risks, including heart disease as part of the AHA questionnaire [14]. Participants were asked how much they worried *(a lot, a little, do not worry at all*) about 12 diseases or risk factors: heart disease/heart attack, AIDS, diabetes, general cancer, breast cancer, stroke, lung cancer, Alzheimer's, osteoporosis, smoking, drug addiction/alcoholism, and violent crime.

**Table 3:** Prevention and knowledge beliefs about heart disease by percent of respondents and education level (*N *= 171)

HD Statement	Agree Strongly	Agree Somewhat	Disagree Somewhat	Disagree Strongly	
When I think of heart disease, I most often think of someone having a heart attack and dying quickly^1^	9.9	44.4	31.0	14.6	Total
	7.8	38.8	35.3	18.1	College
	14.5	56.4	21.8	7.3	No coll.
When I think of stroke, I most often think about someone having a long-term disease that will reduce the quality of their life.	18.7	42.7	22.2	16.4	Total
	19.0	50.9	24.1	18.1	College
	18.2	42.7	18.2	12.7	No coll.
By taking estrogen replacement therapy, I (or women if a male) can help reduce my risk for heart disease.	2.9	47.4	38.6	11.1	Total
	4.3	50.0	34.5	11.2	College
	0	41.8	47.3	10.9	No coll.
I am comfortable talking with my doctor about preventative and treatment options regarding my health.^2^	66.7	20.5	5.8	7.0	Total
	74.1	18.1	2.6	5.2	College
	50.9	25.5	12.7	10.9	No coll.
It is easy to find accurate and easy to understand information about heart disease and stroke.	34.3	50.0	13.4	2.3	Total
	40.5	45.7	12.1	1.7	College
	21.4	58.9	16.1	3.6	No coll.
There is nothing I can do to help prevent myself from getting heart disease. (*HBM Self-efficacy*)^3^	7.0	8.8	11.1	73.1	Total
	5.2	5.2	6.0	83.6	College
	10.9	16.4	21.8	50.9	No coll.
I am at low risk for heart attack or stroke for a person my age. (*HBM Perceived Risk*)	16.9	43.6	27.9	11.6	Total
	18.1	44.0	28.4	9.5	College
	14.3	42.9	26.8	16.1	No coll.

*Note*. Data are % reporting; ^1^p = 0.023; ^2^p = 0.006; ^3^p = 0.000.

Over 50% of the participants expressed that they "did not worry at all" about 7 of these 12 conditions (AIDS, stroke, lung cancer, Alzheimer's, osteoporosis, smoking, drug addiction/alcoholism). Less than 10% worried "a lot" about these conditions with the exception of 17.3% who worried a lot about AIDS. More people worried "a lot" about the remaining five conditions or risks, with over a quarter of respondents naming diabetes as of the greatest concern. In order, the other 4 were, cancer in general, breast cancer, violent crime, and heart disease/heart attack. Women expressed significantly greater concern than men about developing cancer in general (25.8% vs. 8.1%) and breast cancer (27.6% vs. 2.7%). No other significant differences were seen by gender or education.

### Knowledge of risk factors for heart disease

Knowledge of risk factors for heart disease and strategies for prevention are shown in Table 4. Recognition of some risk factors was very high. For example, most participants knew that being overweight (91.9%), having a family history of heart disease (88%), and having high blood pressure (84%) were causes of heart disease. Men in general had higher awareness of many risk factors, including low estrogen (24% vs. 21%) and menopause (18% vs. 15%) than women. As Table 4 shows, college-educated participants had a significantly higher knowledge of risk factors than their peers who were not college educated.

**Table 4:** Percentage of respondents who correctly identified risk factors and prevention strategies (N = 172)

*"Based on what you know, what are the major causes of heart disease?"*					
**Heart Disease (HD) Risk Factors**	**Total**	**Male**	**Female**	**College**	**No college**
		**(n = 62)**	**(n = 110)**	**(n = 116)**	**(n = 56)**

Being overweight	91.9	91.9	91.8	94.0	87.5
Family history of HD	87.8	88.7	87.3	90.5	82.1
High blood pressure	83.7	82.3	84.5	91.4^1^	67.9^1^
Stress	77.3	80.6	75.5	83.6^2^	64.3^2^
High cholesterol	76.7	79.0	75.5	81.0	67.9
Diabetes	68.6	72.6	66.4	69.8	66.1
Not exercising	66.3	69.4	64.5	75.9^3^	46.4^3^
Smoking	62.8	67.7	60.0	62.9	62.5
Racial/ethnic heritage	57.6	61.3	55.5	64.7^4^	42.9^4^
Drinking alcohol	51.2	56.5	48.2	50.0	53.6
Aging	39.5	45.2	36.4	43.1	32.1
Stroke	33.1	24.2	38.2	31.9	35.7
High triglycerides	25.0	29.0	22.7	30.2^5^	14.3^5^
Low levels of estrogen	22.1	24.2	20.9	26.7	12.5
Menopause	15.7	17.7	14.5	19.8^6^	7.1^6^

*"Which of the following do you believe can prevent or reduce the risk of getting heart disease?"*					

Heart Disease (HD) Risk Factors	Total	Male	Female	College	No college
		(n = 62)	(n = 110)	(n = 116)	(n = 56)
Getting physical exercise	93.6	93.4	93.3	94.8	90.9
Losing weight	86.5	90.2	87.5	88.8	81.8
Reducing stress	86.0	86.9	87.5	88.8	80.0
Quitting smoking	84.8	90.2	84.6	81.9	90.9
Reducing sodium or salt in diet	81.9	85.2	86.5	86.2	72.7
Reducing dietary cholesterol	76.6	82.0	76.0	81.0	67.3
Taking vitamins E, C, or A	60.2	70.5	52.9	60.3	60.0
Taking vitamins w/folic acid	43.9	50.8	40.4	49.1	32.7
Reducing animal products in diet	37.4	45.9	40.4	42.2	27.3
Hormone replacement therapy	18.1	24.6	14.4	17.2	20.0
Aromatherapy	14.0	16.4	11.5	15.5	10.9

*Note*. Data are percentage of respondents answering correctly. ^1^p = 0.000; ^2^p = 0.006; ^3^p = 0.000; ^4^p = 0.008; ^5^p = 0.025; ^6^p = 0.043.

Knowledge of ways to prevent or reduce the risk of heart disease was relatively high among all respondents for exercising (94%), losing weight (87%), reducing stress (86%), and quitting smoking (85%). Although 77% of all respondents knew that reducing dietary cholesterol lowered heart-disease risk, only 37% reported that reducing animal products in the diet did the same. A higher percentage of those with a college education than of those without a college education knew of the risk factors (Table 3).

### Knowledge of the symptoms and outcomes of heart disease

A series of 12 true-false statements from the AHA questionnaire determined understanding of heart disease and some behaviors associated with prevention (Table 5) [14]. Several significant differences were observed by gender and education. More women than men knew that heart disease was the leading cause of death among women, that women had atypical symptoms of heart attack, that men and postmenopausal women were equally likely to have heart attacks, and that men were no more likely than women to die from heart disease. Compared with respondents who had a college education, fewer respondents who did not have a college education knew that Black and Hispanic women were more likely than White women to have a heart attack or stroke. Those without a college education also were less aware than those with a college education that there were treatment options to break up blood clots. Women and those respondents with a college education had significantly higher summary index values than men or those who did not have a college education.

**Table 5:** Percent of Respondents Reporting Correctly on Heart Disease Knowledge (HDK) Questions

HDK Statements	Total	Male	Female	College	No College
	(N= 172)	(n= 62)	(n= 110)	(n= 116)	(n= 56)
Some forms of heart disease may result in a stroke. (T)	95.9	95.2	96.4	96.6	94.6
Heart disease develops gradually over many years & can easily go undetected. (T)	92.4	90.3	93.6	93.1	91.1
Women are less likely to get heart disease after menopause than before. (F)	77.3	72.6	80.0	77.6	76.8
In the first few hours after the onset of heart attack or stroke symptoms, treatments exist that can break up blood clots to reduce the damage. (T)	75.4	72.1	77.3	84.5^1^	56.4^1^
Black women are more likely than White women to die from a heart attack or stroke. (T)	73.8	77.4	71.8	81.0^2^	58.9^2^
Hispanic women are more likely than White women to die from a heart attack or stroke. (T)	65.1	66.1	64.5	72.4^3^	50.0^3^
The loss of estrogen is a significant contributor to the development of heart disease in women following menopause. (T)	59.3	56.5	60.9	61.2	55.4
Women are more likely than men to have unusual or atypical symptoms of a heart attack. (T)	54.4	44.3	60.0	58.6	45.5
Men are more likely than postmenopausal women to have heart attacks. (F)	52.9	40.3^4^	60.0^4^	52.6	53.6
Men and women experience the same symptoms of a heart attack. (F)	46.5	41.9	49.1	48.3	42.9
Once men are diagnosed with heart disease, they are more likely than women to become seriously ill or die. (F)	42.4	30.6^5^	49.1^5^	40.5	46.4
Heart disease is the leading cause of death in women. (T)	34.3	12.9^1^	46.4^1^	35.3	32.1
Summary index score	7.7 ± 1.9	7.0 ± 1.9^1^	8.1 ± 1.7^1^	8.0 ± 1.9^6^	7.0 ± 1.7^6^

*Note*. Data are % correct. T = True; F = False. ^1^p = 0.000; ^2^p = 0.002; ^3^p = 0.006; ^4^p = 0.017;^5^p = 0.024; ^6^p = 0.001.

Participants' ability to recognize the warning signs for heart attack and stroke was assessed with 11 questions. The percentage of correct responses for the warning signs of a heart attack were high for chest pain (97%) and tightness in the chest (94%) but low for nausea (30%) and fatigue (50%). Pain spreading to the shoulder, neck, or arms was recognized by 72% of the group overall. For warning signs of stroke, recognition of weakness on one side (85%) and inability to talk or understand speech (80%) were relatively high, but a sudden, severe headache (53%) was less well known. There were no significant differences in knowledge by gender or education. A summary index score was created based on the number of correct answers.

### Self-efficacy to change heart-disease risk

Self-efficacy was evaluated with the statement "There is nothing I can do to help prevent myself from getting heart disease." The majority of respondents (73.1%) strongly disagreed with this statement. Slightly over 11% disagreed somewhat and only 15.8% agreed somewhat or agreed strongly (Table 3).

### Multivariate analysis

Three multivariate linear regression models were developed to determine (a) if differences in CVD knowledge score were predicted by sociodemographic characteristics (gender, age, education) and health status as estimated by number of risk factors (smoking, lack of exercise, overweight/obesity), (b) if perceived susceptibility of CVD risk was associated with gender, age, education, and health status, and (c) if perceived self-efficacy was associated with these same variables. Predictors of the CVD knowledge score included higher education, female gender, high self-efficacy (F = 11.66, adjusted R^2 ^= 0.158, *p *< .001). Higher education and perceived low risk of CVD predicted perceived self-efficacy to heart disease (F = 6.81, adjusted R^2 ^= 0.064, *p *< .001). Male gender, and low self-efficacy were predictors of perceived susceptibility to CVD (F = 4.08, adjusted R^2 ^= 0.035, *p *< .019) (Table 6).

**Table 6:** Significant predictors of heart disease knowledge score, perceived susceptibility to heart disease and perceived self-efficacy

Variable	Beta (p value)	F value	**Adj R**^**2**^	Model p
***Heart Disease Knowledge Score***				
Education Level	.163 (.027)	11.66	.158	.000
Sex	-.249 (.001)			
Self-efficacy to prevent CVD	.223 (.002)			
***Perceived Self-Efficacy***				
Education Level	.226 (.003)	6.81	.064	.001
Perceived low risk of CVD	-.168 (.025)			
***Perceived Susceptibility to Heart Disease***				
Sex	.149 (.049)	4.08	.035	.019
Self-efficacy to prevent CVD	-.148 (.051)			

## Discussion

Our findings demonstrate that some aspects of heart-disease are well known among young adult African Americans. Knowledge of certain other important risk factors and preventive behaviors, however, was more variable and inconsistent among the respondents. There was wide-recognition of the link between being overweight and heart disease. Perhaps more relevant messages and targeted education focused on an area of incongruity in knowledge would resonate better with young adults. One striking observation was that 76.6% of the participants knew that high cholesterol was a major cause of heart disease, but only 37.4% recognized that reducing animal products in the diet is a preventive behavior (Table 4). There appears to be a lack of understanding that high blood cholesterol is associated with consumption of animal products that contain saturated fats. An outreach campaign that focuses on clarification of diet/lifestyle factors that elevate blood cholesterol, including consumption of saturated fat found in animal products would help correct this misperception. These specific differences in awareness need to be considered in the formative evaluation stage of developing health promotion and program planning efforts to a target population [9].

Other researchers have found that knowledge about heart disease differ by gender and education level [34]. Our study supports differences in knowledge by gender, but not always in favor of young adult women. One unexpected finding was that more men than women reported awareness of the effects of low estrogen, menopause, and hormone replacement therapy on heart disease. Women were, however, significantly more knowledgeable than men about women having atypical heart-disease symptoms, increased risk of dying from heart disease, and heart disease as a leading cause of death among this gender group.

Differences in knowledge by education level were more pronounced than those by gender. Although this finding was anticipated, it highlights the importance of tailoring health promotion efforts to specific segments of the targeted population by education level, rather than by ethnicity or gender alone. In some cases, the knowledge gap about a risk factor by education status was quite large. For example, while 91.4% of the respondents who had a college education knew that high blood pressure was a risk factor for heart disease, only 67.9% of the respondents who did not have a college education were aware of this fact. Clearly, more work needs to be done to spread the message about high blood pressure in some parts of the African American community to raise perceived risk and susceptibility awareness.

Another prominent difference between the education-level cohorts was in their reported agreement regarding self-efficacy in prevention of heart disease. Only 51% of those without a college education strongly disagreed that they could not prevent heart disease in contrast to 84% of the college educated (Table 3). A similar divergence was observed with the level of comfort respondents had of talking with a medical doctor. While nearly three-quarters of the college educated strongly agreed they felt comfortable talking with their physician, only slightly over half of the non-college educated felt this way. These differences in responses suggest that persons who were not college educated may feel they have less control over their environment and ability to change risk factors.

Few participants (21%) reported that their physicians had discussed heart disease or its prevention with them. Doctors may not see a need to discuss the issue of heart disease at such an early age if no signs of risk factors are present. While their younger age may indicate lower heart-disease risk, 44% of the group had one risk factor (smoking, engaging in insufficient exercise, or being overweight or obese). Almost 35% of the participants had two or more of these three possible risk factors. The low initiation of CVD discussion by doctors suggests that preventive education could be increased or that respondents were not seeing a doctor regularly. We did not ask about the sources of primary care or insurance status in our study. Some respondents may not have had insurance or a regular physician [35]. Recent analysis of National Health and Nutrition Examination Survey (NHANES) data shows that 59% of young adults (all races) have a CVD risk factor, but screening for cholesterol was less than 50% among these at risk [36]. There may be a role for other contact agencies such as food assistance programs (e.g., WIC) to disseminate sources of CVD prevention information.

The prevalence of overweight or obesity (55%) in our sample population was similar to that of the cohort of African Americans, aged 18-30 years old (57%), in the Coronary Artery Risk Development in Young Adults (CARDIA) study conducted 1990-1991 [27]. Smoking was higher in the CARDIA group (36%), than in the Phoenix sample (8%). The differences in smoking prevalence may be driven by temporal changes in knowledge and behaviors more so than variations in awareness due to regional geography. Because the CARDIA study used open-ended questions whereas our research used a structured questionnaire, the different methodologies in the two studies preclude direct comparison of other CVD knowledge measures.

Overall, there were few differences in risk factors by gender in the sample population. Two notable exceptions were exercise and cholesterol screening. In contrast to their male peers, the young adult African American women in the study were unlikely to engage in physical activity or exercise. Barriers to physical activity for women are essential to identify because barriers are one of the key areas for risk reduction for heart disease, cancer, and diabetes. A positive finding was that 31% of the women had received cholesterol checks in the past 18 months. Since only 16% of men had a cholesterol check, targeted outreach for screening with men is warranted.

The HBM constructs fit well with some of our observations, but there are several inconsistencies within the participant responses. For example, knowledge of some CVD risk factors was quite high, but many participants who knew about these risk factors and had multiple risk factor characteristics did not perceive that they were at risk of CVD. Perceived severity and threat of CVD were relatively low. Self-efficacy for prevention was much higher among those with a college education but was not mediated by the number of risk factors. While the multivariate model for predicting knowledge was relatively strong, the other two models for predicting self-efficacy and perceived risk were not fully explained by the sociodemographic variables. As might be expected from previous studies, young adults did not recognize their own risk status [22,25-28,37].

To prevent heart disease in minority populations such as African Americans, who continue to experience disparities related to heart disease, assessing levels of knowledge about heart disease and initiating preventive measures in young adulthood will help prevention programs succeed. Although knowledge alone may be insufficient to change behavior, assessment of knowledge to develop culturally appropriate messages and determine salient leverage points to encourage behavior change as a first step increases program acceptance [27]. Baseline knowledge assessment as part of all health education and promotion projects during formative evaluation and piloting can make programs relevant, meaningful, and successful with the target audience.

There are several limitations to the study. The participants represent a cross-sectional convenience sample of self-identified, young, adult African Americans. Causality cannot be inferred from the observed associations because of this design. The sample was not random, and findings may not be representative of other young adult African Americans in other Arizona cities or elsewhere. Furthermore, because only African Americans were included in the study, it is not known if these results would be similar or different for same aged people of different ethnic backgrounds.

Despite these limitations, some of the variation observed in knowledge was unexpected. These findings substantiate that learning a health message does not always mean total understanding or the ability to think about it critically. An example is the relatively widespread knowledge that high cholesterol is a known cause of CVD but knowledge that animal products contain high cholesterol is not extensive.

## Conclusions

Heart disease continues to be the leading cause of death for African Americans in Arizona and the rest of the nation, despite decreased death rates during the past several years. Possible tactics to prevent heart disease prevention tactics include increased awareness of risk factors and warning signs. Furthermore, heart disease knowledge could be increased, especially in regard to knowledge of specific risk factors and warning signs of heart attacks in women. The need for increased gender specific knowledge was evident in both male and female participants. By uncovering the reasons that prevent this minority age group from attaining an adequate level of knowledge about heart disease and from changing behaviors when the level of knowledge and health risk are both high, researchers may be able to make a significant contribution to reducing the prevalence of heart disease among young adults.

## Competing interests

The authors declare that they have no competing interests.

## Authors' contributions

DMW and KMJ designed the study together. KMJ conducted the data collection in the field, coordinated the project, drafted the manuscript, and assisted with statistical analysis. DMW revised the manuscript and statistical analysis. Both authors read and approved the final manuscript.

## Pre-publication history

The pre-publication history for this paper can be accessed here:

http://www.biomedcentral.com/1471-2458/11/248/prepub
